# Identification and assessment of PLK1/2/3/4 in lung adenocarcinoma and lung squamous cell carcinoma: Evidence from methylation profile

**DOI:** 10.1111/jcmm.16668

**Published:** 2021-06-02

**Authors:** Sisi Deng, Xiaoli Lu, Zhi Zhang, Rui Meng, Mi Li, Shilin Xia

**Affiliations:** ^1^ Cancer Center Tongji Medical College Union Hospital Huazhong University of Science and Technology Wuhan China; ^2^ Department of Orthopedics Tongji Medical College Tongji Hospital Huazhong University of Science and Technology Wuhan China; ^3^ Shenzhen Huazhong University of Science and Technology Research Institute Shenzhen China; ^4^ Clinical Laboratory of Integrative Medicine The First Affiliated Hospital of Dalian Medical University Dalian China; ^5^ Department of Palliative Medicine Graduate School of Medicine Juntendo University Tokyo Japan

**Keywords:** lung adenocarcinoma, lung squamous cell carcinoma, methylation, PLK1/2/3/4

## Abstract

Lung cancer is a very aggressive cancer characterized with molecular heterogeneities in different subtypes, including lung adenocarcinoma and lung squamous cell carcinoma. However, few related molecular signatures have been established for the treatment of lung cancer subtypes. Polo‐like kinase (PLK) family is a crucial regulator during cell division. Aberrant genetic and epigenetic alteration of PLK members plays a controversial role among different cancers. In this study, we performed an analysis of transcriptional and protein expression to identify overexpressed PLK1/4 and under‐expressed PLK2/3 in lung cancer subtypes. We then analysed biological function of PLKs and related genes. Besides, we estimated a correlation of PLKs with patient's genders and TP53 mutation in lung cancer. Higher PLK1/4 expression was significantly associated with male patient and TP53 mutant status, separately. Moreover, we carried out a methylation profile analysis including methylation level, DNA methyltransferases correlation and survival analysis of global methylation. Global methylation survival analysis showed that prognostic value of PLK1/2/4 methylation remained the same significant trend between two lung cancer subtypes, whereas prognostic value of PLK3 methylation lacked consistency. Taken together, these results provided instructive insights into a comprehensive evaluation for advanced therapeutic strategy based on epigenetic evidences.

## 1 INTRODUCTION

Lung cancer is one of the most refractory malignancies with high incident and mortality. Although the curative effect of lung cancer therapy has improved recent years, its prognosis remains generally poor. The non‐small‐cell lung cancer (NSCLC) comprises >80% of all lung cancer cases and therefore is the focus of lung cancer studies worldwide.^1^ The major pathologic subtypes of NSCLC include lung adenocarcinoma with 40%‐50% proportion and lung squamous cell carcinoma with 25%‐30%.^2^ With great advances in understanding the molecular pathogenesis, lung cancer therapeutic strategy has been switching from a stage‐directed therapeutic pattern to a systematic therapy with histological type and molecular feature.^3^ Initiation and progression of different cancer subtypes is considered a distinct multistep process. An understanding of how genomics alteration and epigenetic modification contribute to tumorigenicity in distinct lung canner subtypes may optimize the lung cancer management.

Precise treatment for lung cancer is dependent on an accurate classification and detailed mechanism of cancer subtypes, otherwise the treatment for lung cancer—not otherwise specified (NOS)—is full of intractable problems. For example, pemetrexed is currently the drug for adenocarcinoma, and bevacizumab is not suitable for squamous cell carcinoma.^4^ Given each subtype of cancer with specific histopathological property, subtype‐specific strategy is predicted to improve a beneficial clinical outcome.

The individual therapy for advanced lung cancer treatment required a better understanding of mechanism not only at histopathological level but also at molecular level, which was because that genomic and epigenetic alteration could drive tumorigenesis of lung cancer subtypes. Prior study has reported that crizotinib is used to target ALK gene rearrangement in NSCLC.^5^ Gefitinib and erlotinib are approved for EGFR mutant status in lung cancer cell.^6^ However, little is known about the clinical settings when distinct lung cancer subtypes cover different molecular alteration.

**FIGURE 1:**
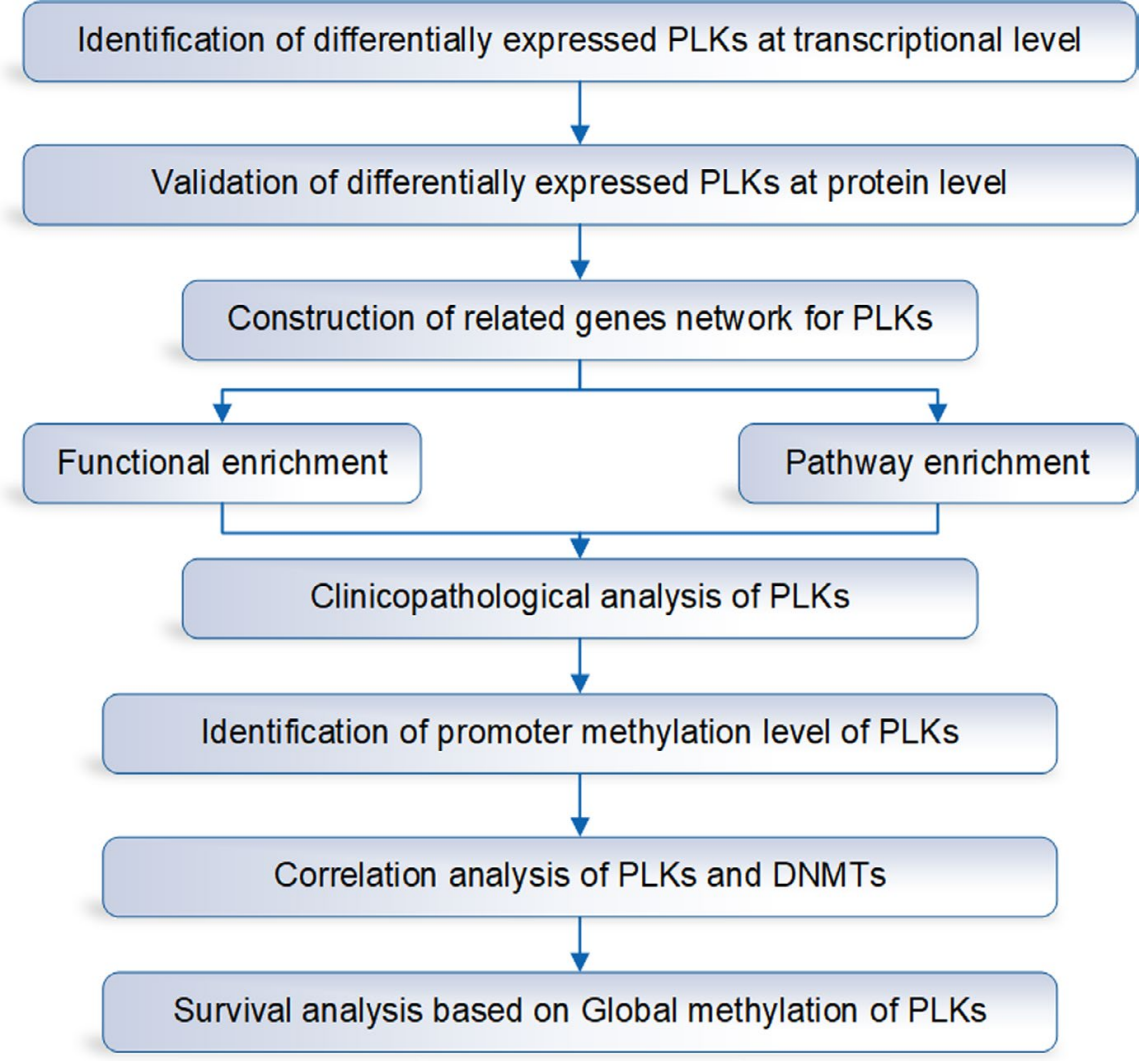
The flow chart of this study based on public databases. Firstly, we identified the differentially expressed polo‐like kinase (PLK)s at transcriptional and protein level. Then, we constructed the related gene network for PLKs for GO and pathway enrichment. Furthermore, we analysed the clinicopathology of PLKs, the promoter methylation level of PLKs, the correlation analysis of PLKs and DNA methyltransferases (DNMTs), and the survival analysis based on global methylation of PLKs

Polo‐like kinase (PLK) family has 5 members, which are implemented in cell cycle regulation and promote subsequent biological process, such as cell division.^7^ It is suggested that PLKs play an important role in cancer cell proliferation. In this study, we identified mRNA and protein expression of PLK family members based on public data platforms, including Oncomine, TCGA, and Human Protein Atlas (HPA) databases. Then, we performed the biological function including GO and KEGG pathway enrichment, as well as a correlation with patient's gender and TP53 mutant status, separately. Furthermore, we evaluated methylation profile of PLK1/2/3/4 including methylation level, DNA methyltransferase (DNMT) correlation and survival analysis of global methylation (Figure 1). These comprehensive analyses in conjunction with clinical data provided an instructive insight into individual therapy for lung cancer subtypes with molecular modification signatures. These molecules may contribute to be targets of future research and potential biomarkers for the lung cancer.

## 2 MATERIALS AND METHODS

### 2.1 Transcriptional expression of PLK1/2/3/4/5 in lung cancer identified in Oncomine database

Oncomine database (http://ualcan.path.uab.edu/index.html) is a bioinformatics initiative to develop a collection for a cancer transcriptome data.^8^ In this study, we analysed the individual gene expression level of PLK1/2/3/4/5 in lung cancer by Oncomine. The mRNA levels of PLK1/2/3/4/5 were compared between lung cancer and normal patient data sets. The threshold in our study was 1.5‐fold change, *P*‐value = .0001 and top 10% gene rank.

### 2.2 Transcriptional expression of PLK1/2/3/4 in both lung cancer subtypes validated by TCGA database

UALCAN (http://ualcan.path.uab.edu/index.html) is an interactive web‐portal for providing the in‐depth analyses of TCGA gene expression data.^9^ TCGA consortium has enabled clinical researchers and cancer scientists to investigate the molecular characteristics and expression profile of gene across various cancers. The relative expression of PLK1/2/3/4 was analysed between cancer and normal samples in lung cancer subtypes including lung adenocarcinoma and lung squamous cell carcinoma. A *P*‐value less than .05 is statistically significant.

### 2.3 The protein expression patterns of PLK1/2/3/4 based on immunohistochemistry

The HPA (https://www.proteinatlas.org) is a web tool which provides protein localization and expression in human tissues.^10^ The result of immunohistochemistry was generated by unique antibody application. In the current study, the immunohistochemistry images were collected to show an overview of PLK1/2/3/4 between cancer and normal tissue in lung adenocarcinoma and lung squamous cell carcinoma, respectively. In our collection, antibodies for PLK1/2/3/4 in lung cancer were HPA053229, CAB009624, HPA060318 and HPA035026.

### 2.4 Construction of related gene network for PLK1/2/3/4 in relation to biological function and pathway

STRING version 11.0 (https://string‐db.org/) integrates publicly available sources to generate connectivity network of protein‐protein association.^11^ The proteins of interest were uploaded to achieve a global network in order to analyse physical and functional interaction. Here, the network of PLK1/2/3/4‐related genes was performed via STRING with confidence score = 0.400.

GeneMANIA (http://www.genemania.org) is a prediction server for biological function of input genes from a wealth of genomics and proteomics data.^12^ Based on a large set of functional association data, input gene was conducted to show functional association network. We submitted PLK1/2/3/4 to perform an interactive network among PLK1/2/3/4 and their related genes. The data were automatically weighted to calculate an association with the advanced statistical options including max resultant genes = 20 and max resultant attributes = 10.

WEB‐based Gene SeT AnaLysis Toolkit (WebGestalt; http://www.webgestalt.org) is a platform for facilitating a comprehension of functional enrichment.^13^ In this study, the method of interest in over‐representation analysis (ORA) was implemented. DAVID (https://david.ncifcrf.gov/summary.jsp), as a resource of functional annotation,^14^ was used to enrich KEGG pathway of PLK1/2/3/4 and their 20 related genes, as well as GO enrichment analysis including cellular component (CC), biological process (BP) and molecular function (MF).

### 2.5 The association of PLK1/2/3/4 expression with genders, TP53 mutation in both lung cancer subtypes

Form April 2020, UALCAN (http://ualcan.path.uab.edu/index.html) initiated an analysis of gene expression profile based on TP53 mutation status. Given the modulation relationship between PLK1/2/3/4 and TP53, we assessed the association of PLK1/2/3/4 expression between TP53 mutant and TP53‐notmutant in lung adenocarcinoma and lung squamous cell carcinoma.

In addition, UALCAN is used to depict the clinical features of genes including various clinical information. UALCAN in our study was utilized to compare the transcriptional expression of PLK1/2/3/4 between male and female in lung adenocarcinoma and lung squamous cell carcinoma. The *P*‐value less than .05 was considered as statistically significant.

### 2.6 Methylation analysis of PLK1/2/3/4 genes in both lung cancer subtypes

Given the different expressions of PLK1/2/3/4 genes between lung cancer and normal tissue, we carried out a methylation analysis in UALCAN web to evaluate epigenetic regulation of PLK1/2/3/4 expression by promoter methylation. A *P*‐value less than .05 showed a statistical significance.

The cBio Cancer Genomics Portal (cBioPortal; http://cbioportal.org) is a web resource to exhibit a multidimensional investigation for cancer genomics data.^15^ Based on TCGA data, DNA methylation value was released and added to the portal. The mRNA expressions of DNMT1/3A/3B were correlated with the mRNA expression of PLK1/2/3/4 in lung adenocarcinoma and lung squamous cell carcinoma. Spearman and Pearson correlation were used to measure the association between DNMT1/3A/3B and PLK1/2/3/4 in both lung cancer subtypes. The *P*‐value less than .05 was considered as statistically significant.

MethSurv (https://biit.cs.ut.ee/methsurv/) is a web tool to perform multivariable survival analysis based on CpG methylation patterns.^16^ MethSurv utilizes TCGA methylation data to provide an overview analysis of methylation differences. In the present study, survival probability of patients with lung cancer was assessed using likelihood‐ratio (LR) test to evaluate global DNA methylation survival of PLK1/2/3/4 in lung adenocarcinoma and lung squamous cell carcinoma.

## 3 RESULTS

### 3.1 Transcriptional expression of PLK1/2/3/4/5 in patients with lung cancer

In order to assess expression pattern of PLK family members in lung cancer, we compared the transcriptional levels of PLK1/2/3/4/5 with those in normal samples in Oncomine database (Table 1 and Figure S1). The PLK1 was significantly overexpressed in patients with lung cancer in six data sets. In Hou lung Statistics,^17^ the most samples among six data sets, PLK1 expression was increased in lung adenocarcinoma with a fold change of 2.115 and in lung squamous cell carcinoma with a fold change of 2.741. In contrast to PLK1 expression, PLK2/3 showed an under‐expression in lung cancer compared to normal sample. In Bhattacharjee Lung Statistics,^18^ PLK2 was found to be lower expressed in small cell lung carcinoma (fold change = −5.625) and lung carcinoid tumour (fold change = −7.189) versus normal sample. Hou et al^17^ reported that PLK3 expression was decreased in lung adenocarcinoma (fold change = −2.348) and in lung squamous cell carcinoma (fold change = −1.890). Compared to normal samples, PLK4 was found to be higher in lung cancer sample.^19^ While compared to normal sample, PLK5 showed no differential expression either in lung adenocarcinoma or in lung squamous cell carcinoma. As a result, our findings indicated that PLK family members had distinct expression characteristics in lung cancer.

**TABLE 1:** Transcription expression of polo‐like kinase (PLK) family members between lung cancer and normal lung tissues (Oncomine)

	Types of lung cancer vs lung	Fold change	*P*‐value	*t* test	Ref
PLK1	Lung adenocarcinoma	2.115	1.05E‐16	11.397	Hou Lung
	Lung adenocarcinoma	2.348	4.99E‐06	4.873	Su Lung
	Lung adenocarcinoma	3.127	2.63E‐05	4.593	Stearman Lung
	Squamous cell lung carcinoma	2.741	4.75E‐15	13.452	Hou Lung
	Squamous cell lung carcinoma	5.076	4.27E‐05	7.556	Garber Lung
PLK3	Squamous cell lung carcinoma	−2.348	4.28E‐15	−9.878	Hou Lung
PLK4	Squamous cell lung carcinoma	2.415	1.64E‐12	10.595	Hou Lung

### 3.2 Identification of PLK1/2/3/4 expression in two subtypes of lung cancer

**FIGURE 2:**
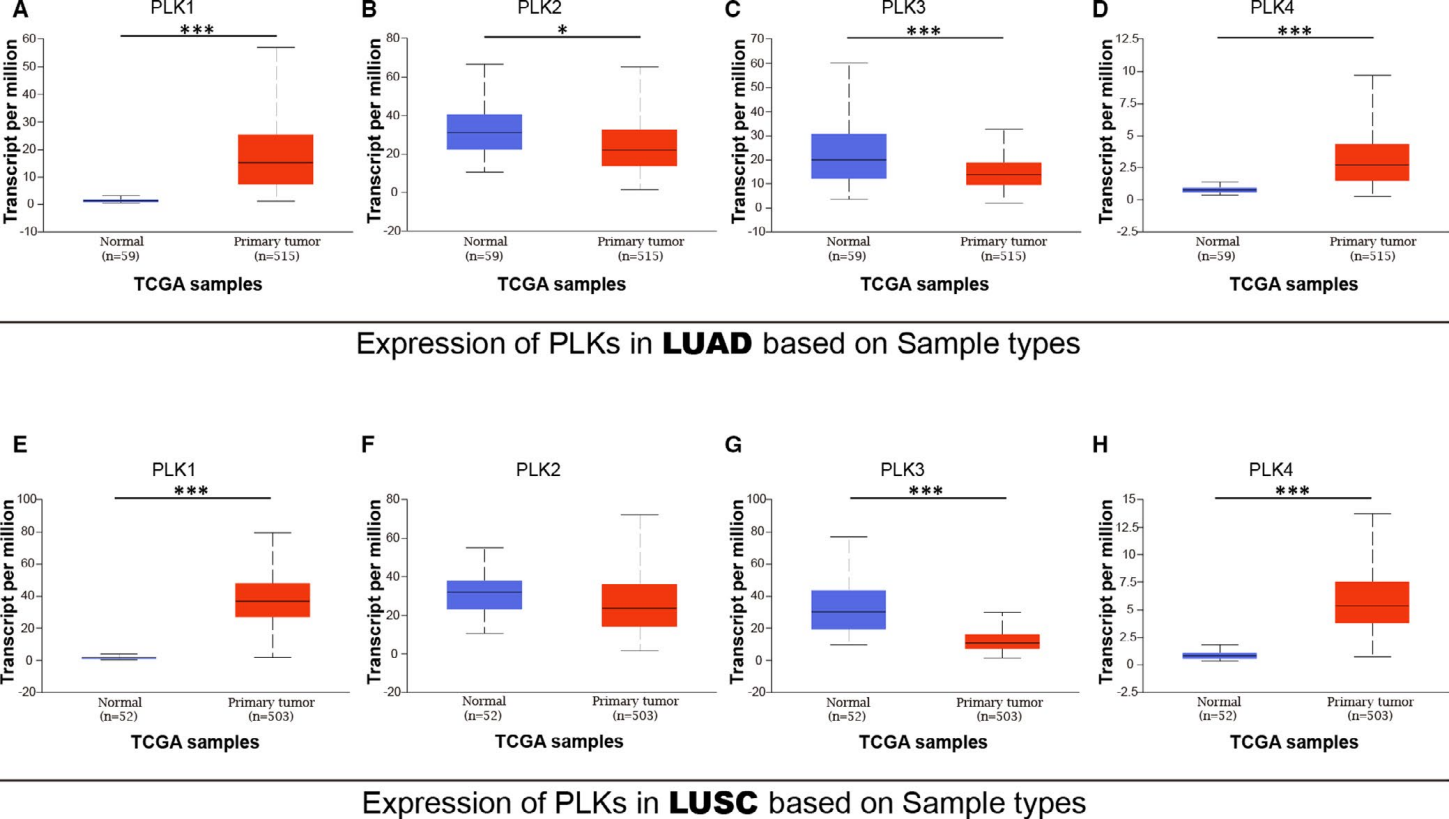
Transcriptional expression of polo‐like kinase (PLK)1/2/3/4 between lung cancer subtypes and adjacent normal tissues. Transcriptional expressions of PLK1/4 were found to be overexpressed in primary lung adenocarcinoma tissues compared to normal samples (A‐D). Transcriptional expressions of PLK1/4 were found to be under‐expressed in lung squamous cell carcinoma tissues compared to normal samples (E‐H). *P < .05 and ***P < .001. LUAD: lung adenocarcinoma and LUSC: lung squamous cell carcinoma

Given that we found the differential expression of PLK1/2/3/4 in lung cancer, further identification of PLK1/2/3/4 expression pleads for an observation of their expression in lung cancer subtypes, including lung adenocarcinoma and lung squamous cell carcinoma. In lung adenocarcinoma (Figure 2A‐D), PLK1/4 were overexpressed in primary tumour from 515 lung adenocarcinoma compared to 59 normal samples (*P* < .001). PLK2 expression was lower in lung adenocarcinoma (*P* < .05), and PLK3 was also under‐expressed in lung adenocarcinoma (*P* < .001).

In lung squamous cell carcinoma (Figure 2E‐H), PLK1/4 were found higher expressed in primary tumour from a comparison between 503 primary tumour samples and 52 normal samples (*P* < .001). The data from PLK2 expression revealed a slight decline in primary lung squamous cell carcinoma (*P* = .146). PLK3 was found lower expressed in lung squamous cell carcinoma than normal sample. Overall, these results in two lung cancer subtypes from TCGA corroborated the findings of PLK1/2/3/4 expression from Oncomine database.

### 3.3 Protein expression patterns of PLK1/2/3/4 in lung cancer subtypes

**FIGURE 3:**
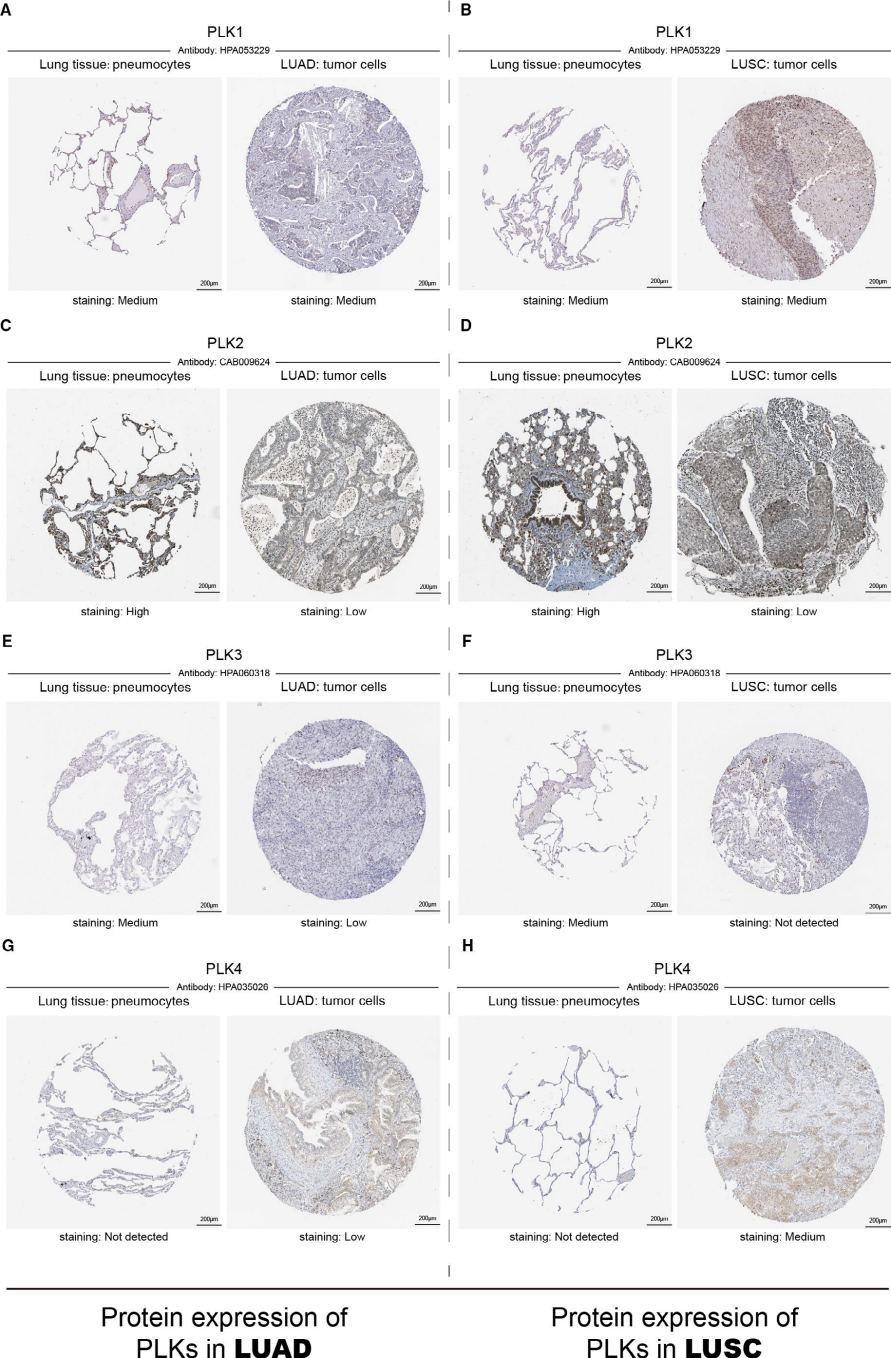
Immunohistochemistry result of polo‐like kinase (PLK)1/2/3/4 between lung cancer sample and pneumocytes of lung tissue. Immunohistochemistry images of PLK1/2/3/4 were displayed to show PLK protein expression patterns in lung adenocarcinoma and lung squamous cell carcinoma. Antibodies for PLK1/2/3/4 in both lung cancer subtypes were HPA053229, CAB009624, HPA060318 and HPA 035026. LUAD: lung adenocarcinoma and LUSC: lung squamous cell carcinoma

After examining the transcriptional expression of PLK1/2/3/4 in lung cancer, we continued to compare the protein expression patterns of PLK1/2/3/4 in both lung adenocarcinoma and lung squamous cell carcinoma. As shown in immunohistochemistry image from HPA platform, PLK1 protein showed a differentiation in neither lung adenocarcinoma nor lung squamous cell carcinoma (Figure 3A,B). The staining of PLK2 protein was found high in both subtypes of lung cancer with a low level in normal tissue (Figure 3C,D). PLK3 was higher expressed in pneumocytes of lung tissue than that in either lung adenocarcinoma or lung squamous cell carcinoma (Figure 3E,F). In normal lung tissue, PLK4 protein was not detected but at low level in lung adenocarcinoma and medium level in lung squamous cell carcinoma (Figure 3G,H). In summary, these immunohistochemistry images basically supported that PLK1/2/3/4 proteins were in accordance with their transcriptional expression in lung cancer.

### 3.4 Biological function and KEGG pathway of PLK1/2/3/4 and their related genes

**FIGURE 4:**
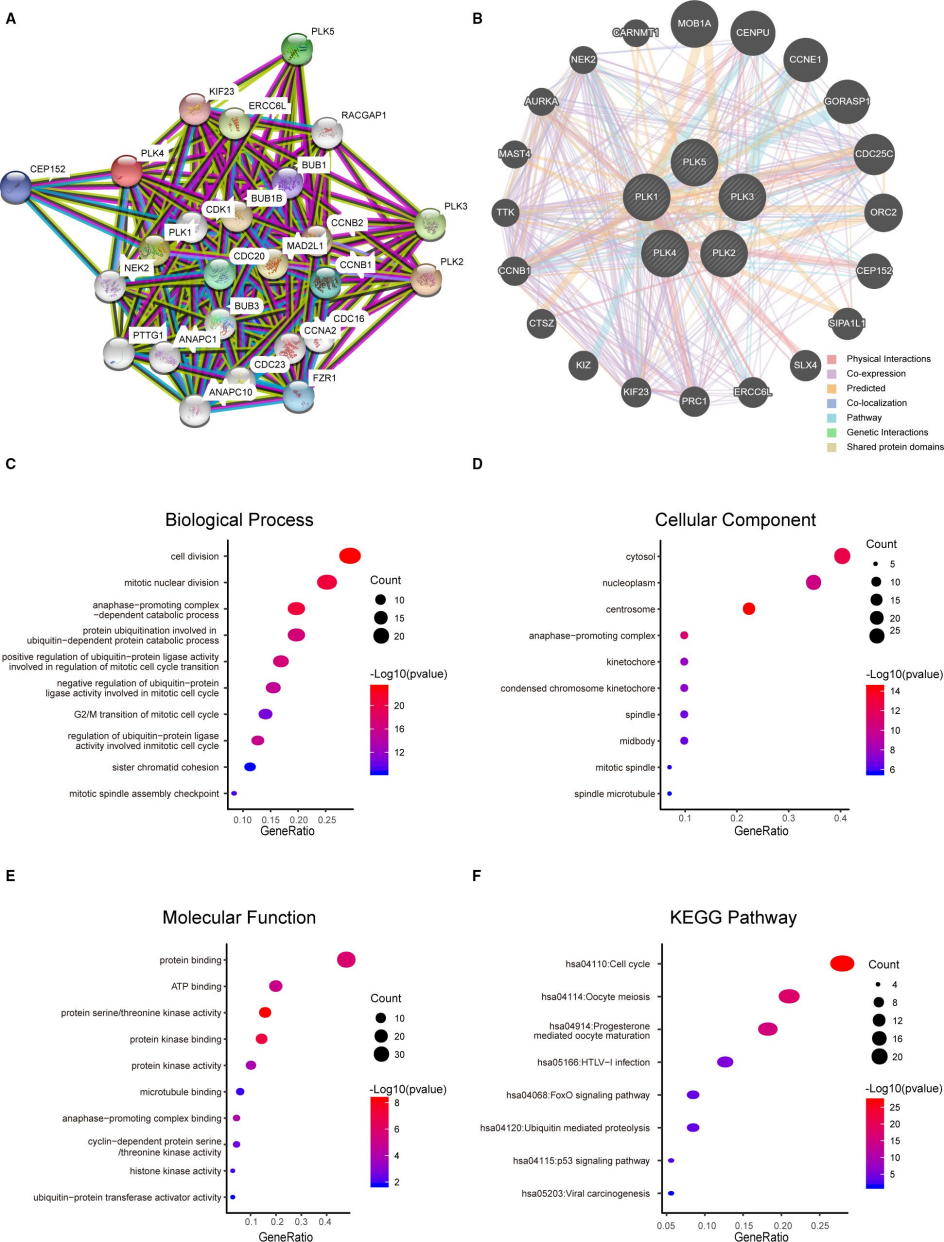
PPI network, biological functions and KEGG pathways of polo‐like kinase (PLK)1/2/3/4 and their related genes. PPI network was constructed to present a connectivity of PLKs and their related genes (A, B). GO functional enrichment was performed to show main biological functions of PLK1/2/3/4 and their related genes, including biological process, cellular components and molecular functions (C‐E). KEGG pathway of PLK1/2/3/4 and their related genes was enriched (F)

Since PLK1/2/3/4 exhibited differential expression in lung cancer subtypes, we carried out to a biological analysis including protein‐protein interaction, GO and KEGG pathway enrichment. The PPI network of PLK1/2/3/4 with their 21 related genes was obtained by STRING followed by a visualization of GeneMANIA (Figure 4A,B). The primary interaction comprised physical interactions, co‐expression, prediction and co‐localization.

GO function analysis was enriched including biological process (BP), cellular component (CC) and molecular function (MF). In biological process, the majority of 25 genes were associated with cell division, mitotic nuclear division and anaphase‐promoting complex‐dependent catabolic process (Figure 4C). In cellular component, it was obviously found that cytosol, nucleoplasm and centrosome were the most component involved with PLK1/2/3/4 as well as their related genes (Figure 4D). In molecular function, the proteins of these molecules mainly engaged in the binding function including protein binding and ATP binding (Figure 4E). According to the KEGG pathway analysis, cell cycle and oocyte meiosis were the most two pathways involved by these 25 molecules (Figure 4F). Thus, we concluded that the function of PLK1/2/3/4 and their related genes were primarily related to cell division and cell cycle.

### 3.5 The correlation of PLK1/2/3/4 with patient's gender and TP53 mutant status in lung cancer subtypes

**FIGURE 5:**
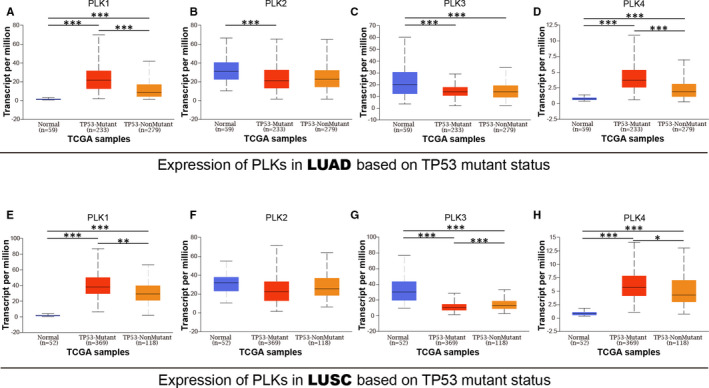
Correlation between transcription expression of polo‐like kinase (PLK)s and TP53 mutant status in lung cancer. A total of lung adenocarcinoma sample included 59 normal, 233 male and 279 female samples. A total of lung squamous cell carcinoma sample included 52 normal, 369 male and 118 female samples. Transcription expressions of polo‐like kinase (PLK)1/2/3/4 were correlated with TP53 mutant status in lung adenocarcinoma (A‐D) and lung squamous cell carcinoma (E‐H) *P < .05, **P < .01 and ***P < .001. LUAD: lung adenocarcinoma and LUSC: lung squamous cell carcinoma

Due to the oocyte meiosis pathway involved by PLK1/2/3/4 and their related genes, we further explored a correlation of PLK1/2/3/4 with TP53 mutation. In both lung cancer subtypes, significantly higher expressions of PLK1/4 were found in TP53 mutant lung cancer than that in TP53‐wide subtypes (Figure 5A,D,E,H). PLK2 expression was not related with TP53 mutation in either of lung cancer subtypes (Figure 5B,F). PLK3 presented a lower expression in TP53 mutant lung squamous cell carcinoma. Taken together, our results showed that PLK1/3/4 expressions were remarkably associated with TP53 mutant status in lung cancer subtypes.

Next, we aimed to distinguish the differential expression of PLK1/2/3/4 between genders of patients with lung cancer subtypes. PLK1 expression was higher in male than that in female patients (Figure S2A,E), so was PLK4 expression between genders (Figure S2D,H), whereas PLK2 presented no differential expression between male and female of lung cancer patients (Figure S2B,F). PLK3 was higher expressed in female than male only in lung adenocarcinoma (Figure S2C,G). These results indicated that PLK1/3/4 played a differential role between genders of patients with lung cancer subtypes.

### 3.6 The methylation analysis of PLK1/2/3/4 in lung cancer

**FIGURE 6:**
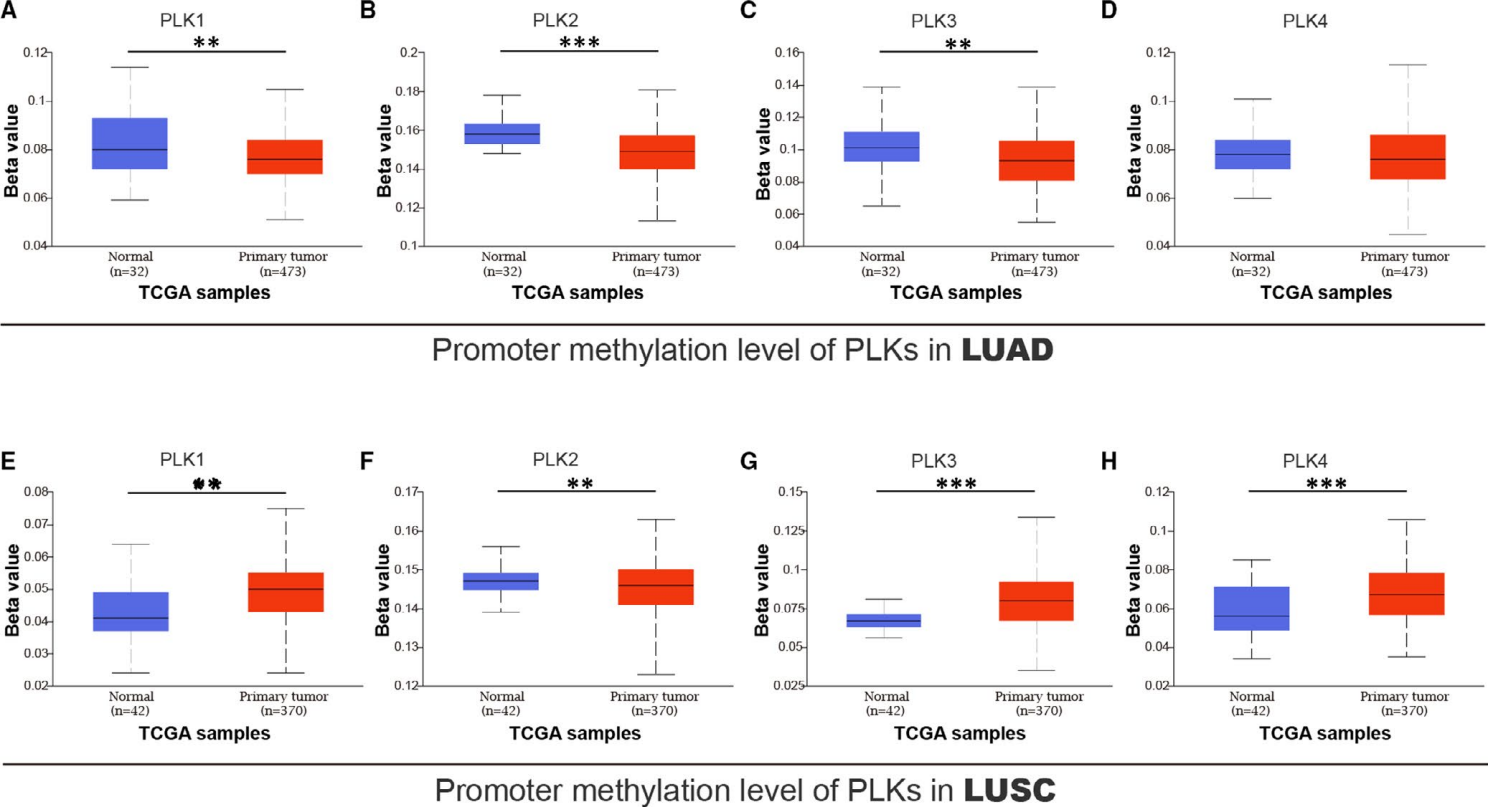
Promoter methylation of polo‐like kinase (PLK)1/2/3/4 in lung cancer. A total of lung adenocarcinoma sample included 32 normal and 473 primary tumour samples. A total of lung squamous cell carcinoma sample included 42 normal and 370 primary tumour samples. Promoter methylation of PLK1/2/3/4 was analysed in lung adenocarcinoma (A‐D) and lung squamous cell carcinoma (E‐H) **P < .01 and ***P < .001. LUAD: lung adenocarcinoma and LUSC: lung squamous cell carcinoma

To explore an epigenetic mechanism driving the differential expression of PLKs in lung cancer, we investigated the promoter methylation level of PLK1/2/3/4 in lung adenocarcinoma and lung squamous cell carcinoma. In lung adenocarcinoma, PLK1/2/3 promoters had a lower level in primary tumour than those in normal tissue (Figure 6A‐C), and PLK4 promoter showed no significant alteration between normal and tumour primary (Figure 6D). In lung squamous cell carcinoma, PLK1/3/4 promoters presented a higher methylation in primary tumour, whereas PLK2 promoter methylation was at lower level in primary tumour than that in normal sample.

After an observation of methylation in PLK promoters, we performed a correlation between PLK1/2/3/4 and DNMT1/3A/3B. DNMT1/3A/3B were positively related with PLK1/4 in lung cancer subtypes (Figure S3), except the correlation between DNMT3A and PLK1 without significance in lung squamous cell carcinoma (Figure S3E). In lung adenocarcinoma, DNMT3A/3B had a negative correlation with PLK3 (Figure S3B,C). In lung squamous cell carcinoma, DNMT1 expression was negatively related with PLK2 level (Figure S3D). Our findings revealed the correlation between mRNA levels of PLKs and DNMTs, providing a new insight into epigenetic regulation of PLK1/2/3/4 expression in lung cancer subtypes.

### 3.7 Prognostic value of global methylation of PLK1/2/3/4 in patients with lung cancer subtypes

**FIGURE 7:**
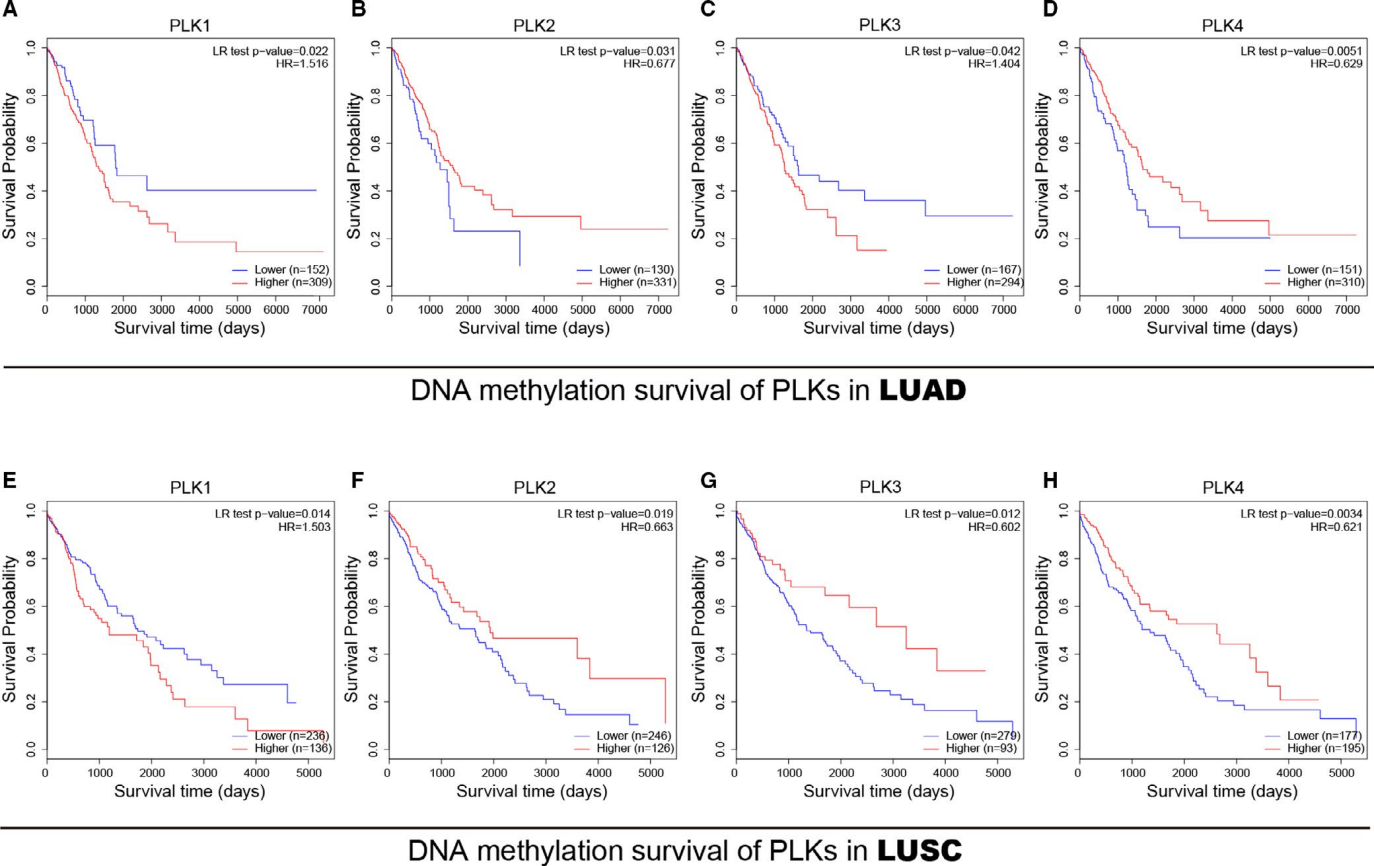
Prognostic value of transcriptional expression of polo‐like kinase (PLK)1/2/3/4 in liver cancer patients. Survival time (days) of PLK global methylation was evaluated in lung adenocarcinoma (A‐D) and lung squamous cell carcinoma (E‐H). The *P*‐value less than .05 was considered as statistically significant. LUAD: lung adenocarcinoma and LUSC: lung squamous cell carcinoma

Based on the methylation analysis, we further analysed the prognostic values of global methylation of PLK1/2/3/4 in lung adenocarcinoma and lung squamous cell carcinoma. Higher methylation level of PLK1/3 promoter was associated with poor survival probability of patients with lung adenocarcinoma (Figure 7A,C), while higher methylation level of PLK2/4 was significantly related to favourable survival probability of patients with lung adenocarcinoma (Figure 7B,D). In lung squamous cell carcinoma, PLK2/3/4 methylation was dramatically associated with longer survival time of lung cancer (Figure 7F,G,H), whereas PLK1 methylation was significantly associated with shorter survival time (Figure 7E). Our results indicated that global methylation of PLK1/2/3/4 was obviously associated with lung cancer patients' prognosis and they may be exploited as potential targets for lung cancer therapy.

## 4 DISCUSSION

Being serine/threonine protein kinases, PLK family proteins perform several important functions throughout cell cycle, which may drive a malignant cancer under abnormal modulation. Despite PLK expression is found to be regulated by epigenetic regulation in some malignancy, the mechanism underlying epigenetic patterns remains to be addressed in lung cancer, especially distinct subtypes. Previous studies show that aberrant methylation regulation plays an oncogenic role in development and progression of lung cancer.^20^ In this study, the methylation profile of PLK1/2/3/4 was analysed in lung adenocarcinoma and lung squamous cell carcinoma, as well as prognostic value of PLK promoter methylation in lung cancer subtypes.

Our results presented that overexpression of mRNA and protein was found in PLK1/4 and under‐expression of mRNA and protein was found in PLK2/3. Since that PLKs and related genes were found to be implicated in oocyte meiosis pathway, we estimated a correlation between PLKs and gender of patients with distinct lung cancer subtypes, separately. Higher mRNA expressions of PLK1/4 were significantly related to male patients with either of lung cancer subtype, and higher mRNA expression of PLK3 was apparently associated with female patients with lung adenocarcinoma. We continued to measure a correlation between PLK expression and TP53 mutant status. Methylation analysis displayed that methylation of PLK1/2/3 genes remained a higher level in normal lung sample than that in lung adenocarcinoma. The methylation of PLK1/3/4 genes kept a higher level in lung squamous cell carcinoma sample than that in normal lung sample; however, PLK2 methylation stayed at lower level in lung squamous cell carcinoma than normal tissue. Multivariate analysis provided evidences which reflected a correlation between mRNA expressions of DNMT1/3A/3B and PLK1/2/3/4 and the prognostic value of PLK methylation.

Given that methylation alteration participates in massive expression regulation for cancer progression, we investigated a methylation profile of PLK gene in lung cancer.

DNMT1/3A/3B, with the DNA methyltransferase catalytic activity, rewrite the epigenetic landscape of multigene. We observed that PLK1/2/3/4 methylation in lung adenocarcinoma was inferior to that in normal sample. While in lung squamous cell carcinoma, the methylation was strengthened in PLK1/3/4 genes while weakened in PLK2 gene. Accordingly, we observed that the transcriptional expression of DNMTs had a positive relation with PLK1/4 and a variously negative association with PLK2/3 in lung cancer subtypes. These results indicated that methylation of PLK2/3 genes may be regulated by DNMTs, whereas the methylation mechanism of PLK1/4 remains bleak. One possible reason is that distinct DNMT expression is mediated by post‐transcriptional, translational or post‐translational modifications. The other reason is that DNMT catalyse methylation site would not impact on PLK transcription. In addition, global methylation survival analysis showed that prognostic value of PLK1/2/4 methylation kept a similar trend between two lung cancer subtypes, whereas prognostic value of PLK3 methylation showed different between subtypes. Results from prognostic evaluation showed that global methylations of PLKs were putative prognostic signatures for survival probability of patients with lung cancer. These findings provided a preliminary insight into the prognostic value of epigenetic modification, such as methylation pattern.

PLK1, a serine/threonine protein kinase belonging to CDC5/Polo subfamily, plays a promotive role during mitosis.^21^ As mentioned in the literature reviews, PLK1 regulates centrosome maturation and assemblies the bipolar spindle.^22^ PLK1 is also known as an important component for removing the cohesins from chromosome arms. Since PLK1 is involved in the regulation of mitotic exit and cytokinesis, PLK1 expression has been found with a high level in various cancers.^23^ Disruption of PLK1 mitotic pathway in lung adenocarcinoma cells dramatically inhibited cell proliferation.^24^ A report about NSCLC showed that PLK1/cMet axis mediated apoptosis of NSCLC cell.^25^ Another prior study presented an antitumour activity of PLK1 inhibitor in NSCLC.^26^ In the current study, PLK1 expression was elevated in lung adenocarcinoma and lung squamous cell carcinoma, which was consistent with the findings of a great deal of previous work. Because of oocyte meiosis pathway mediated by PLK1, we found that transcriptional expression of PLK1 decreased in female patients with either of lung cancer subtypes, which indicated that PLK1‐mediated pathway preferred to facilitate a development of lung cancer in male patients. The mutation of TP53 gene, as a tumour suppressor gene, is a canonical oncogenetic factor among diverse malignancies. Previous studies evaluating P53 observed inconsistent results on PLK‐regulated pathway.^27^ We found that PLK1 expression was increased with TP53 mutant status, indicating that cancer occurrence and development gained from a drive from synergistic function between TP53 mutation and PLK1 activity in lung adenocarcinoma and lung squamous cell carcinoma.

PLK2 is one of polo family of serine/threonine protein kinases, which influenced cell division.^28^ Serum can enhance an expression of PLK2, suggesting that PLK2 plays a potential role in undergoing rapid cell division.^29^ PLK2 was regarded as contradictory role in cancer development. On the one hand, PLK2 was reported as tumour suppressor to promote cancer growth and inhibit apoptosis in colorectal cancer,^30^ osteosarcoma^31^ and cholangiocarcinoma.^32^ On the other hand, some findings pointed that down‐regulation of PLK2 suppressed apoptosis of laryngeal carcinoma^33^ and gastric cancer cell.^34^ The role of PLK2 in lung cancer remains inconclusive. We analysed differential expression pattern by Oncomine, TCGA and HPA, which demonstrated that the mRNA and protein level of PLK2 were lower in lung cancer than that in normal sample. In accordance with methylation level and the correlation with DNMTs, PLK2 expression was not intervened by methylation in lung adenocarcinoma or lung squamous cell carcinoma.

Similar to PLK1/2, PLK3 was involved in regulation of cell cycle progression,^35^ and it acted as a cancer suppressor in several types of malignancies.^36^ Some results from in vitro and in vivo studies showed that PLK3 inhibited the growth of colorectal cancer.^37^ Lv et al^38^ reported that PLK3 suppressed the proliferation of osteosarcoma cell. However, the biological role of PLK3 in lung cancer is still limited. Wiest et al^39^ found that PLK3 expression is down‐regulated in a majority of lung carcinoma samples. The results in this study supported Wiest's findings that PLK3 expression was decreased in lung cancer. mRNA expression of PLK3 significantly increased in lung adenocarcinoma than that in normal sample, which was therefore likely that mRNA level of PLK3 would be a biomarker for male patient with lung adenocarcinoma. Moreover, PLK3 was negatively correlated with TP53 mutant status in lung squamous cell carcinoma. Further validation needs to discuss the association between TP53 mutation and PLK3.

As a member of polo family of serine/threonine protein kinases, PLK4 localizes to centrioles and regulates centriole duplication during the cell cycle.^40^ PLK4 was identified as overexpressed kinase in multiple malignancy, such as high‐grade glioma.^41^ PLK4 promoted cell proliferation and tumorigenesis of glioblastoma.^41^ PLK4 overexpression improved cell proliferation and invasion of hepatocellular carcinoma.^42^ However, to date, little is known regarding the role of PLK4 in lung cancer. In the current study, although PLK4 was overexpressed in lung cancer, methylation could not explain the transcriptional and protein expression of PLK4. Like the role of PLK1 between genders, high expression of PLK4 was associated with male patient. An understanding of how PLK1/4 contributes to tumorigenicity of lung cancer in male patients may enable their use as a therapeutic target for distinct gender patients. The same trend of PLK1/4 was observed in the analysis of TP53 mutant status, which also pleaded for a solid evidence to investigate the PLK1/4 and TP53 mutation.

To gain more insights into the methylation profile of PLKs, we performed a multivariate analysis based on in silico data. However, the main weakness of this study was the paucity of concrete data from in vivo and in vitro experiments to validate our findings, such as the prognostic value of global methylation pattern of PLKs. Another limitation is that large sample size is required in future studies to explore clinical features of PLKs, such as stages or grades. The detailed mechanism is worth to be investigated for the integrated prognostic role in lung cancer subtypes.

In summary, we herein identified the transcriptional and protein expression of PLK1/2/3/4 in lung adenocarcinoma and lung squamous cell carcinoma. After biological function analysis, we estimated the correlations of PLKs with patient gender and TP53 mutant status, separately. Subsequently, we further evaluated methylation profile of PLKs, including methylation level, DNMT correlation and survival analysis of global methylation. In both subtypes of lung cancer, these results indicated that epigenetic signature like methylation profile could be exploited as a cluster of prognostic biomarkers for survivals of lung cancer patients. Our study revealed the function of PLK protein family in lung cancer, especially the relationship between the methylation of PLK family and the survival of patients, which is a new perspective for further research. These results can provide new theoretical support for improving the prognosis of lung cancer patients.

## CONFLICT OF INTEREST

The authors declare that they have no competing interests.

## AUTHOR CONTRIBUTIONS

**Sisi Deng:** Data curation (lead); Writing‐original draft (lead); Writing‐review & editing (lead). **Xiaoli Lu:** Formal analysis (equal). **Zhi Zhang:** Formal analysis (equal). **Rui Meng:** Formal analysis (equal). **Mi Li:** Conceptualization (equal); Data curation (equal). **Shilin Xia:** Conceptualization (equal); Data curation (equal).

## Supporting information

Figure S1

Figure S2

Figure S3

## Data Availability

The data that support the findings of this study were generated at TCGA, CPTAC and HPA. Derived data supporting the findings of this study are available from the corresponding author on reasonable request.
