# Supplementary figures and images for: Identification and assessment of PLK1/2/3/4 in lung adenocarcinoma and lung squamous cell carcinoma: Evidence from methylation profile
Source: J Cell Mol Med. 2021 Jun 2;25(14):6652–63. doi: 10.1111/jcmm.16668 (PMC8278123; doi:10.1111/jcmm.16668)

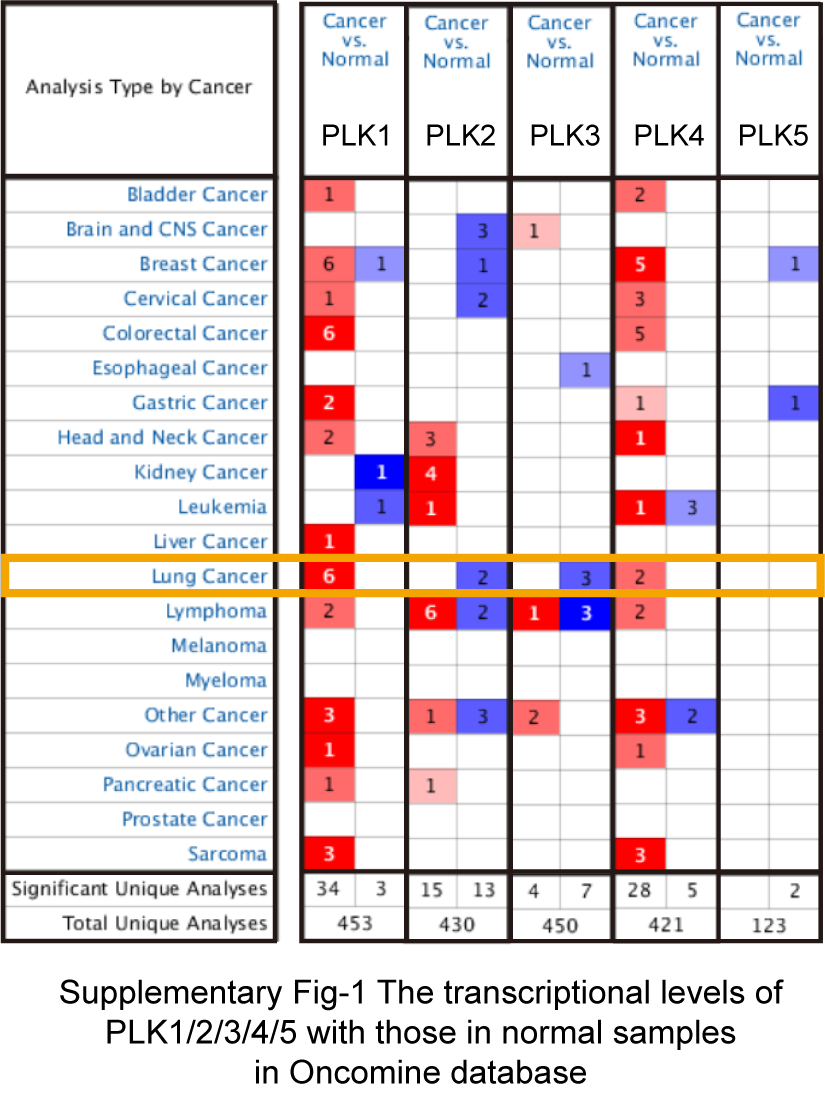

Supplement: Supplementary file 1 — Figure S1 [file JCMM-25-6652-s001.tif]

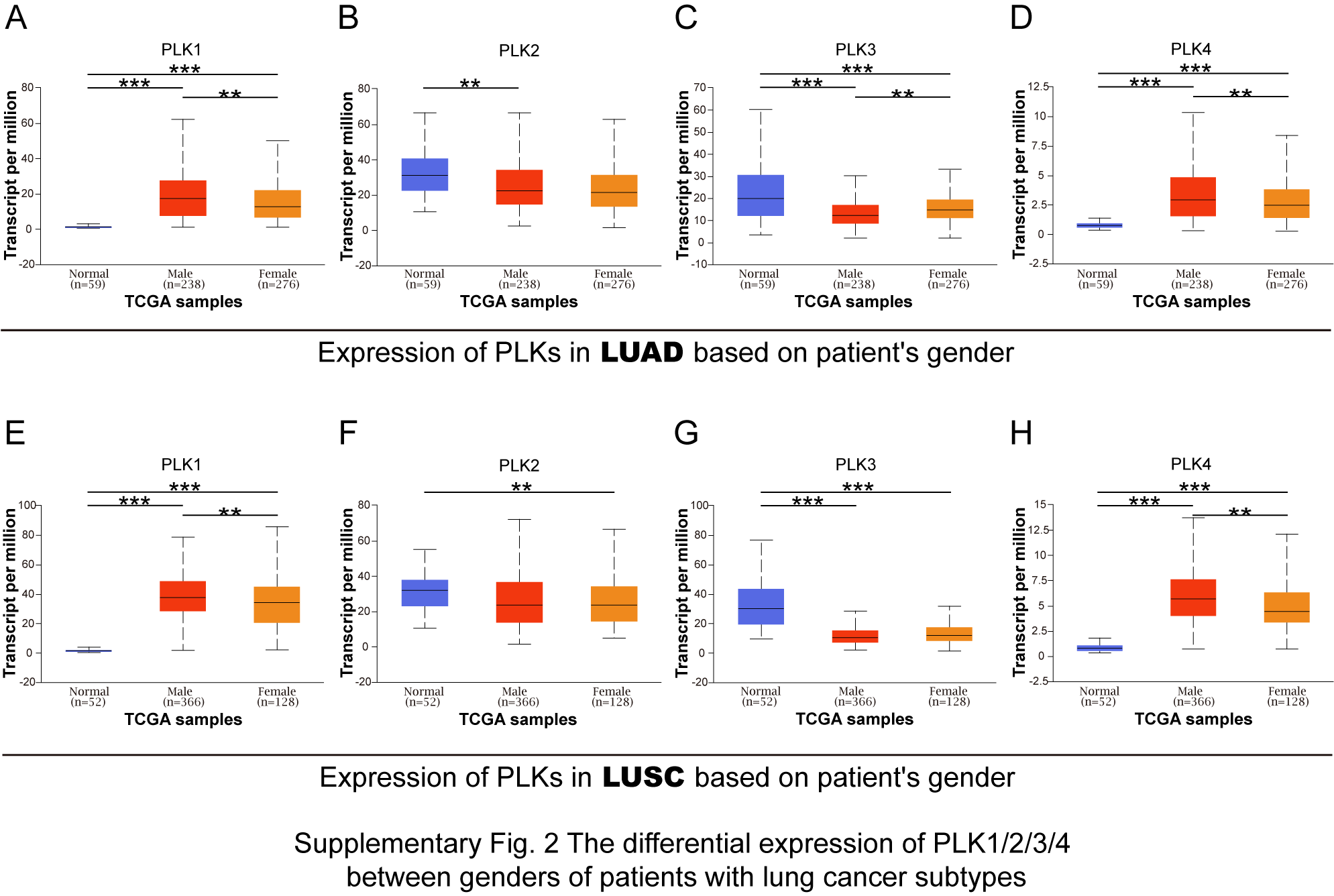

Supplement: Supplementary file 2 — Figure S2 [file JCMM-25-6652-s002.tif]

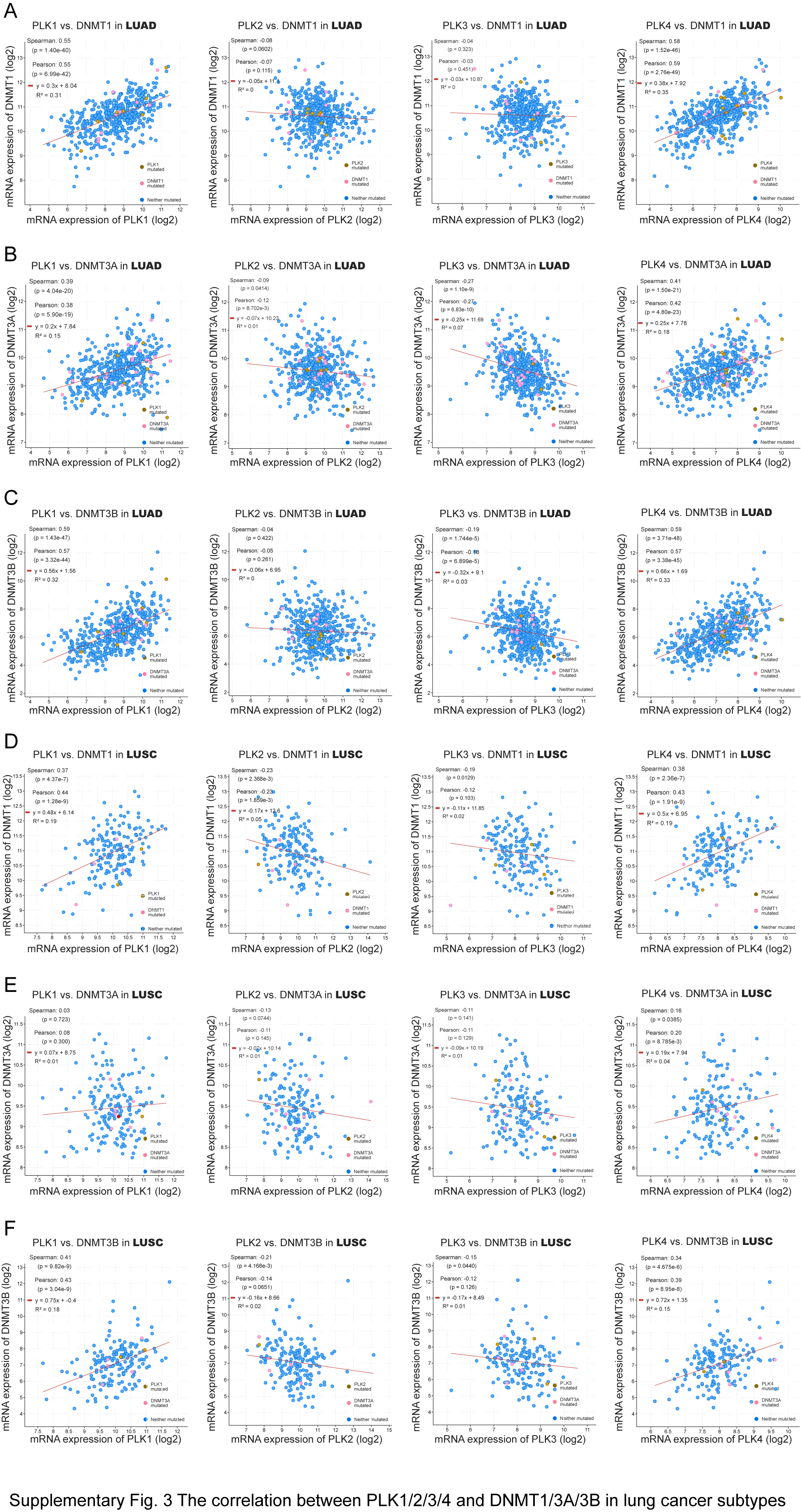

Supplement: Supplementary file 3 — Figure S3 [file JCMM-25-6652-s003.tif]
